# Oral cancer knowledge in adults evaluated through a phone survey in the context of the SARS-CoV2 health emergency in Colombia

**DOI:** 10.4317/medoral.26031

**Published:** 2023-08-25

**Authors:** Iris Lucía Espinoza, Yuliana Elizabeth Serna, María Camila Fuentes, Andrea Jaramillo, Karen Tatiana Piedrahita, Gloria Jeanethe Alvarez

**Affiliations:** 1 DDS, MSc, PhD, associate professor, School of Dentistry, Universidad de Chile, Chile; 2 Dental student, School of Dentistry, Universidad de Antioquia. Medellín, Colombia; 3 DDS, Specialist in Stomatology and Oral Surgery. Full professor, School of Dentistry, Universidad de Antioquia. Medellín, Colombia

## Abstract

**Background:**

in Colombia, oral cancer is the eighth most common type of cancer, with an estimated survival rate of 52%. Lack of knowledge about oral cancer and its risk factors is associated with late detection.

**Material and Methods:**

a descriptive cross-sectional study was carried out on 268 patients attending the School of Dentistry of the University of Antioquia clinics, to whom a validated 47-question questionnaire was applied by phone during the COVID-19 pandemic (2020 and 2021). Data were analyzed using the SPSS software.

**Results:**

the mean age was 58.9. 58.6% of the participants had heard of oral cancer, 42% acquire knowledge from the media, and 96.7% considered screening necessary. Most of the patients expressed not feeling worried (54.5%), fearful (59.7%), or anxious (56.3%) in the case of being submitted to an examination for early detection of oral cancer. A correlation between low socioeconomic status and educational level with less knowledge of oral cancer was found. The dimensions of experience and attitude towards screening were not associated with sociodemographic variables.

**Conclusions:**

There is little knowledge about oral cancer, especially in low socioeconomic and educational status, although this does not occur in the dimensions of attitude and experience toward screening. In contrast, patients participating in this study presented high knowledge about oral cancer risk. This population recognizes the situations most related to the development of cancer. This level of knowledge was similar for the demographic conditions except for people with no education, who presented less knowledge of the risks. The need for educational campaigns on oral cancer knowledge is reaffirmed, especially in socially disadvantaged groups, considering that there would be no barriers related to screening.

** Key words:**Oral cancer, knowledge, adults, COVID-19 pandemic.

## Introduction

Incidence and mortality rates of head and neck cancer in Central and South American regions vary considerably between countries, with the highest rates observed in Brazil, Cuba, French Guyana, Uruguay and Argentina (1). By 2020, cancer of the lip and oral cavity ranks 16th in incidence and mortality worldwide (2). In Colombia, a recent study with the information of Cali city shows an incidence of head and neck cancer of 6.1/100000 inhabitants, ranking 11th among all types of cancer; the most frequent location is tongue and the histological type is squamous cell carcinoma, with 77.2% of frequency (3). One of the most significant problems with oral cancer in Colombia is its poor survival after diagnosis, with a 36.38% of lethality estimated (4).

One of the factors related to poor survival is the delay in the initiation of treatment that, in the first weeks after the onset of oral cancer, is due to late consultation by patients (5). Studies in different countries reveal that lack of knowledge about the signs and symptoms of oral cancer and its risk factors (6-10) may be associated with the initial late consultation. The COVID 19 health emergency declared by the government of Colombia in 2020 and the lockdown caused delayed in consultations and probably could influence the oral cancer awareness in the population. However, we did not find studies on knowledge of oral cancer in Colombia reported in indexed literature during this period.

For that reason, we consider it is important to study oral cancer knowledge and associated variables in adult patients treated in the Dental Clinics of the University of Antioquia, Medellin, Colombia. The results will provide information that allows the design of educational programs to reduce the lack of knowledge, improve prognosis, life expectancy, and reduce morbimortality. In the context of the COVID-19 pandemic, the recommendations of the health emergency restricted dental care. This forced us to seek new strategies for undertaking this research such as the application of a phone survey.

## Material and Methods

A descriptive cross-sectional study was carried out in a population composed of patients over 18 years of age, attending the undergraduate clinics of the last semesters of the School of Dentistry of the University of Antioquia as well as the clinics of the postgraduate programs of orthodontics, periodontics, and oral rehabilitation of the same institution.

For the sample size calculation, we took into consideration the variable “response to the proportion of knowledge of oral cancer” (Have you heard of oral cancer?). Our previous indexed literature review, between the years 2002-17, included nine studies, and have determined that the proportion of positive responses to this question is between 23.7% - 91.2%, with a proportion of 66%. Considering that the average number of patients seen in the selected clinics for this study was 3683 during 2018 and the confidence level (Z) and precision or maximum allowable error in terms of proportion (d) were respectively 95% and 0.05, the minimum required sample size based on the prevalence estimation (frequency of knowledge of oral cancer in the population) was 274. An oversampling of 32% was considered for possible losses due to the use of the phone survey and the pandemic context of COVID-19. 362 phone calls were made to patients between March 2020 and February 2021.

Inclusion criteria included patients over 18 years of age who agreed to participate in the study and the exclusion criteria were patients with a history of oral cancer. 88 individuals refused to participate due to data protection, fear, or lack of time, and 6 patients were excluded due to a history of oral cancer, leaving a final sample of 268 patients.

- Instruments

Oral cancer knowledge and perception:

A validated 47-question questionnaire (11) including a linguistic adaptation was applied. Items related to the Colombian population were also incorporated. Those were: the use of vaping, inverted smoking, and aguardiente (a typical liquor). The instrument was divided into 5 sections: 1. sociodemographic data (age, gender, marital status, ethnicity, and educational level); 2. use of health services; 3. knowledge and attitudes about oral cancer (risk factors, existence of precursor lesions, professionals that would be consulted in case of suspicion of oral cancer, clinical examination, among others); 4. lifestyle (habits) and 5. occupational aspects. The sociodemographic and knowledge variables were categorized for descriptive analysis and evaluation of possible relationships. The questionnaire was applied by phone due to the COVID-19 health contingency and the participant verbally accepted the informed consent. The questionnaire was administered by 4 previously trained undergraduate dental students.

Statistical analysis:

Data were collected in a google form and imported into Excel for data cleaning and quality assessment. A complete response was obtained for all the questions associated with the instrument.

The analyses included a univariate descriptive approach to describe the absolute and relative frequencies of demographic variables, history of alcohol and cigarette consumption, and clinical history and oral cancer knowledge. Bivariate analyses were performed to analyze the answers to the questions "have you heard of oral cancer?" and "do you know about oral cancer?". Regarding the demographic variables, the chi-square test for association or trend was applied.

The items of oral cancer knowledge and perception scale were summarized into dimensions based on the model presented by Awojobi (11). Therefore, they were grouped into 1) screening experience, 2) attitude toward screening, 3) risk knowledge, and 4) emotion. For each dimension, a total score was calculated by adding the items; some items were inverted to verify correspondence to a single directionality of the dimension. For the scores, summary statistics were applied: mean, standard deviation, median, maximum, and minimum.

Finally, cross-tabulations were constructed to explore the relationship between the demographic variables and the scores of the dimensions of interest previously mentioned. The mean with the 95% confidence interval was reported, along with the minimum and maximum scores. For all bivariate analyses, 95% confidence and 5% statistical significance were estimated. The analyses were performed in SPSS version 27.

## Results

- Sociodemographic characteristics, use of health services, and habits of participants

The mean age of the participants was 58.9 with a range between 21 and 86 years. Of the 268 participants, 71.3% were women, 63.1% belonged to the middle socioeconomic stratum, 43.7% were married and 62.3% stated that they did not belong to any ethnic group. Secondary schooling predominated in 61.2% of the population and 86.9% of the participants had worked at some time in their lives (Table 1).

The jobs were grouped according to the single classification of occupations for Colombia. The most reported were officers, operators, artisans, and related trades workers with 25.2%, which include, among others, garment makers and carpenters, followed by elementary occupations such as housekeepers with 20% and, in third place professionals, scientists, and intellectuals with 19.6%.

**Table 1:**
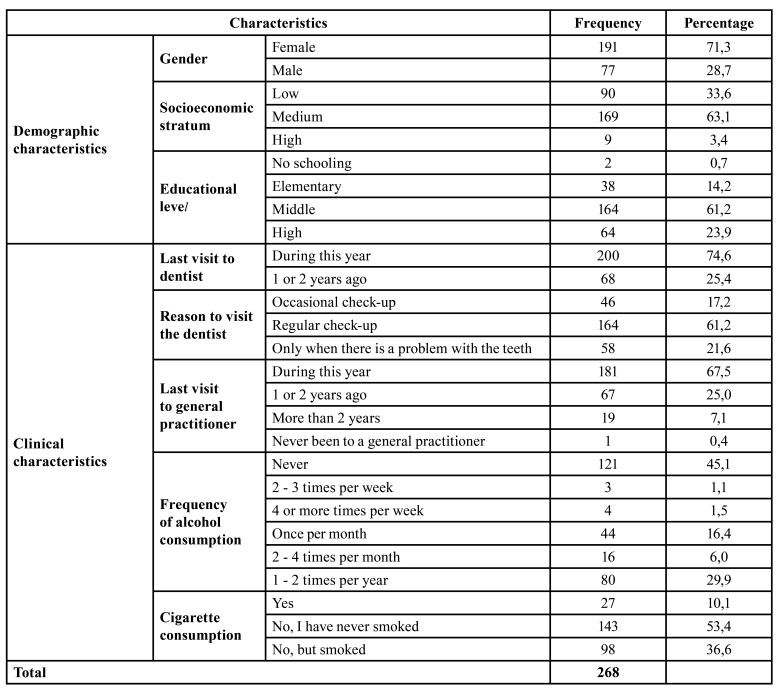
Sociodemographic characteristics, use of health services, and habits of the participants.

- Use of health services and habits

Regarding the use of health services, 67.5% of the respondents reported having attended to see a physician and 74.6% to a dentist in the last year. While the main reason for visiting the dentist is the regular check-up (61.2%), 21.6% attend only when they have a problem with their teeth (Table 1).

53.4% of the participants stated that they had never smoked. The population that consumed other types of tobacco, such as chewing tobacco, represented 1.1% of the total sample. A total of 45.1% of the subjects reported never having consumed alcohol. Among the consumers, aguardiente was the most consumed liquor (26.8%), followed by beer (24.2%) and Ron (18.8%).

- Oral cancer knowledge and attitudes

58.6% of the participants have heard of oral cancer, and when asked ¿how much do you think you know about this topic? only 1.9% mentioned knowing a lot and 35.4% said they knew nothing at all (Table 2). 42% of those who had heard about oral cancer obtained the information from the media (including television, radio, internet, posters, advertising on cigarette packs, and YouTube), 37.6% from friends or relatives, 5.7% within their academic training at school or university, 6.4% from health professionals, 2.6% at work, and the remaining 5.7% obtained from various sources of information.

On the other hand, 48.5% stated that they had never been screened for early signs of oral cancer; however, 94% would like their dentist to inform them if they have been screened for these signs (Table 2).

**Table 2:**
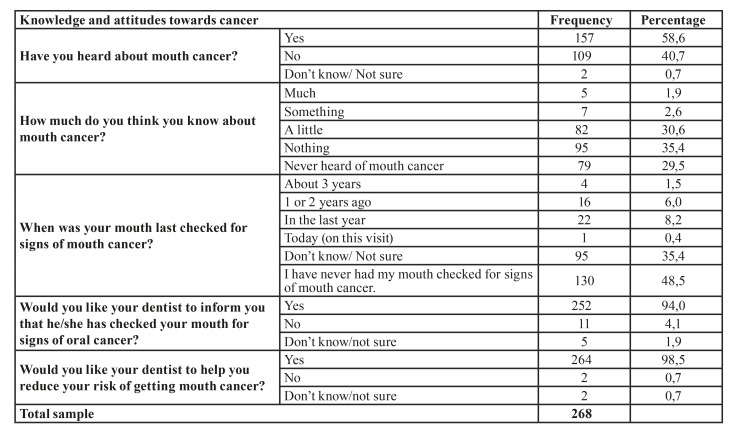
Knowledge and attitudes regarding oral cancer.

40.7% of the participants consider that those trained to detect oral cancer are general dentists and specialists, 32.5% think that those are dentists, physicians, and dental students, 15.3% said they are general practitioners, and specialists, 10.8% do not know or are not sure which professional can detect it, and the remaining percentage consider those are other professionals. Only 17.2% of those surveyed know of the specialty of oral pathology.

About 50% would go to the general practitioner for signs such as red spots, yellow spots, lumps or swelling, and pain, and the other 50% of the patients would go to the dentist. However, for the sign of white spots in the mouth, 49.3% of people would consult a dentist and 46.3% would consult a general practitioner. A minimal percentage of patients would go to the pharmacist or other health personnel for the care of these symptoms.

A total of 96.7% of the patients thought that screening for oral cancer is necessary, 73.5% mentioned that their dentist will not cause them discomfort during the examination, and 91% considered that they will be given an early diagnosis of oral cancer if they have it. It was evident that 64.6% of those surveyed believe that this examination does not hurt, 63.4% said that it only takes a few minutes, 88.4% mentioned it should be done regardless of whether or not the person has dental prostheses, and 94.4% considered that it is a way of discovering this disease early and it should be done in all people regardless of age (95.1%). Additionally, 74.3% of those surveyed thought that this examination is performed using X-rays. Most patients expressed not feeling worried (54.5%), fearful (59.7%), or anxious (56.3%) in the event of undergoing routine screening for oral cancer.

- Oral cancer risk factors, knowledge, and lifestyles

Of the 268 respondents it was found that people identified the main risk factors for oral cancer as cigarette smoking (89.6%), inverted smoking (84.0%), and chewing tobacco (70.1%). More than half of the population associated it with the use of vapes (61.2%) as well as alcohol consumption (60.4%). 85.2% considered that labeling campaigns on cigarette packs have not had an impact on reducing consumption.

- Relationship between dimensions

The scores were calculated by adding the items. The score of the experience of screening dimension (discomfort, duration, early detection) ranged from a minimum of zero to a maximum of eight points, with a mean score of 4.5 (Standard Deviation (SD) 1.5). The attitude score (positive) towards screening presented a mean score of 3.7 points (SD 1.3), this score ranged from zero to seven points. Knowledge of risks presented a score that ranged between five and fifteen points, with a mean score of 11 points (SD 1.96). Finally, in the emotion dimension (feeling apprehension, fear, or concern) the mean score was lower (2.62; SD 3.8), this score ranged between zero and fifteen points.

A significant correlation was found between individuals who had heard about oral cancer and the socioeconomic stratum to which they belonged, where the majority of those in the middle stratum (*p*=0.014) had heard at least once in their life about this disease. Likewise, most respondents who completed secondary education and/or had higher education (*p*=0.048) had heard and were aware of the existence of oral cancer, unlike those who had not studied.

There was a significant correlation (*p*=<0.0001) between knowledge of cancer and the education level of the population. Which, most of the participants who mentioned knowing a lot or a little about cancer (60%) had higher education and most of the participants who mentioned knowing nothing or a little about this topic only completed their middle or high school.

Table 3 shows the behavior of the scores of each dimension according to selected demographic variables. All the mean scores of the dimensions were similar among the three socioeconomic levels, except for the emotion dimension score, which was lower in the high stratum (0.8, 95% CI -0.1, 1.6). Generally, among the men in the study, mean scores were higher than those found in the women, with variations of one to two points with respect to the latter group of participants. However, in the emotional dimension, women had the highest mean score (2.9, 95% CI 2.3 - 3.4). Among respondents over 60 years old, the highest mean score for screening experience was found to correspond to 4.6 points. In contrast, among adults aged 41 to 60 years, the highest mean scores for attitude toward screening [3.8] and knowledge of risk [11.2] were observed.

**Table 3:**
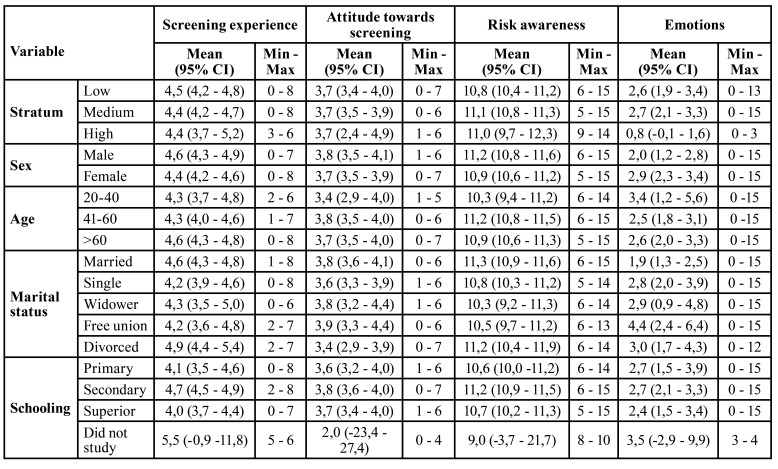
Relationship between sociodemographic variables and the dimensions of screening experience, attitude towards screening, knowledge, and emotions.

Among young people and adults aged 20 to 40 years, a mean score of 3.4 points was observed in the emotional dimension, which was the highest in comparison with participants of other ages. Among participants with no schooling, the highest scores were found for screening experience [5.5] and emotion [3.5]. On the other hand, the population with high school education showed higher scores for attitude toward screening and knowledge of risk, with 3.8 and 11.2 points, respectively.

## Discussion

The results presented in this study show that knowledge about oral cancer and its risk factors as well as the emotions toward a screening examination are directly related to socioeconomic status and educational level. This study, carried out in the context of the COVID-19 pandemic, applied a validated survey by performing phone interviews (11). To our best knowledge, this study is the first one to use this strategy. There were two factors that facilitated its execution; first, the population included is captive since they belong to the dental clinics of the School of Dentistry of the University of Antioquia, and second, the participating individuals were willing to participate in the phone survey from the first contact. However, as a weakness, we can mention that the results cannot be extrapolated to the general population because the individuals were patients that were treated in the clinics of the school.

According to the sociodemographic data obtained, there is a greater participation of women in this study. It has been shown that women attend more often to health services and tend to respond more to survey-based studies, a situation that is also observed in various studies in countries such as Brazil (12).

More than half of the population in this study recognizes the existence of oral cancer (58.6%), a finding that agrees with several studies worldwide (5-9,13-15) with percentages of knowledge ranging between 22% and 94%. Although it is lower than reported in some countries, the level or depth of knowledge is low. Table 4 shows percentage and characteristics of oral cancer knowledge in different populations. This highlights the need for public awareness and education programs to increase knowledge about oral cancer.

Knowledge acquired about this disease was through media (42%), friends or relatives (37.6%), and very little comes from the health professional. Similar findings were reported in other studies such as those conducted by Prado (12) and Bajracharya (16). On the other hand, a significant correlation was found between individuals who have heard of oral cancer and their socioeconomic stratum. The majority correspond to the middle stratum, to which most participants of the study belong, since they have greater access to education and health services (6), as was also demonstrated here, with a significant correlation between knowledge of cancer and higher educational level. While this finding is in line with research conducted in other countries (12,16), it is in contrast with the research conducted by Panzarella (10) in diagnostic delay in oral squamous cell carcinoma, where no correlation was found.

As in most studies (6,7,9,12,16), knowledge of risk factors such as tobacco use in all its forms, alcohol consumption, and although the evidence is not yet conclusive, it is interesting how respondents also associate the use of vapes and, to a lesser extent, alcohol. In the case of smoking, it is important to note that people report that strategies such as campaigns via cigarette packs have not influenced the reduction of consumption (8.9%), unlike what was reported by Zachar (7) in Australia; although it confirms that the media such as television, internet, and social networks play an important role in the education about oral cancer and risk factors. But, the health professional does not exert an important influence on it.

**Table 4:**
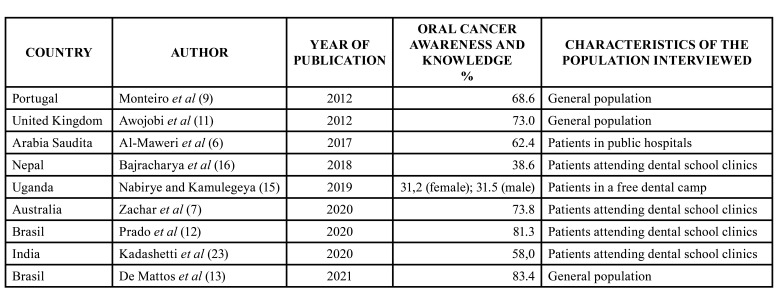
Oral cancer awareness and knowledge in general population or patients in dental clinics/hospitals in different countries.

In Colombia, prevalence of tobacco consumption was 9.8% in 2019 (17). Despite this decrease in consumption in the population, and the fact that most respondents were able to associate tobacco consumption with oral cancer as in the world literature (18), 37.7% of the participants mentioned having consumed cigarettes at some time in their lives.

It is noteworthy that most of the respondents (73.1%) place the use of dentures as a risk factor and exclude gender or age. According to several studies (18,19), a significant correlation exists between chronic trauma caused by poor-fitting dental dentures and oral cancer.

This study did not ask about other known risk factors such as sun exposure and Human Papilloma Virus (HPV). Although the association of oral cancer with HPV has not yet been established, it is in the case of oropharyngeal cancer.

Regarding oral self-examination, few participants are aware of its existence as well as the clinical dental examination's existence for oral cancer detection (5). This finding is similar to the observed in the study reported by Awojobi (11) with 14%.

It was found that the participants do not differentiate between the health professional (physician and dentist) that is trained to recognize early signs of oral cancer. Therefore, they would either go to the former or the latter. Although, there is a slight tendency to go mainly to the physician. Awojobi (11) observed that participants reported that "they would visit their doctor rather than their Dentist for individual signs of oral cancer" and "they did not know if their Dentist screens them for signs of oral cancer as part of their routine check-up”. This is confirmed in this study, where almost half of the participants reported never having been screened for oral cancer, demonstrating little recognition of the dentist's role in oral mucosal examination and oral cancer detection, and confirming the secondary role given to the dentist in early detection (5). Possibly, the limited access to dental health services, and the scarcity of knowledge about dentists’ skills for early detection, added to deficiencies in the professional's knowledge about oral cancer, can explain this situation. Therefore, it is necessary to reinforce the knowledge of the population about the role of the dentist and how he/she contributes to the early detection, diagnosis, and treatment of oral cancer. This should be done, among others, by the dental professional himself, during the visit, when examines clinically, in a systematic way, and informs that is performing a screening for oral cancer (20,21). This is confirmed by this study, where most of the respondents want their dentist to inform them if they have been examined for signs of oral cancer, they consider that the examination is necessary and facilitates early diagnosis, i.e., the population is interested and has a positive attitude towards this type of screening. However, it is not known whether the patients have been examined for oral cancer or whether they have simply not been informed, so it is necessary to investigate this matter among dentists and dental students.

On the other hand, the management of emotions associated with oral cancer screening (anxiety, fear, or concern) was good. In general, patients indicated that they would act calmly when faced with an examination for oral cancer. The mean score was similar to the study conducted by Awojobi (2.62 and 2.52 respectively) (11). Because in our study some items were inverted to correspond to a single directionality of the dimension, the attitude towards screening or the experience of undergoing the examination, such as recognizing if it hurts, understanding the duration, or if it causes discomfort, among others, the mean score was lower [3.75] than the reported by Awojobi [13.4] and did not show a clear trend among key characteristics of the population such as age, sex, among others. The need for educational campaigns on oral cancer awareness is reaffirmed, especially in socially disadvantaged groups, considering that there would be no barriers regarding attitudes and emotions about screening.

In this population, it is noteworthy that the majority of individuals felt that the use of X-rays is necessary for oral cancer screening, which could be related to the routine use of this diagnostic aid (panoramic radiography) during the first appointment at the School of Dentistry.

The present study provides evidence on the knowledge and attitudes of patients who consult a dental school regarding oral cancer, the implications of sociodemographic aspects such as the social status and educational level (6), and confirms the need to carry out educational campaigns regarding the early signs and symptoms of this disease, the risk factors and the professionals trained for its detection, which can be evaluated later and verify that they contribute to the early detection of oral cancer and therefore to greater survival. All this should be done hand in hand with the professional and the mass media to achieve greater dissemination of information.

The relevance of doing this study during pandemics is to know if there is any impact of the pandemic on these topics of the population as an important area of study of oral cancer. Some studies in cancer patients during pandemics have found that their attitudes to lockdown measures were positive, and other studies, have found that these patients have suffered from low quality of life due to inadequate to meet their care needs and higher levels of risk perception associated with stress. However, the pandemic does not seem to impact the results of this survey, but it is necessary to interview new individuals after pandemics and compare if there would be any difference in the results (21,22).

Among health professionals, dentists, and dental schools are a very important source of education. Therefore, they must provide information on oral cancer and its risk factors as well as guidance on prevention in routine consultations. These institutions and dentists could play a more active role in social networks, which are, currently, one of the most influential educational media, especially for young people (23). However, due to the importance of the medical personnel as the first professional to be consulted, it would be a priority the incorporation of an oral cancer course in the medical curriculum and continuing education for this group.
